# Gatekeepers to Justice: Police Officers’ Experiences Responding to Sexual Assault

**DOI:** 10.1177/00111287221143944

**Published:** 2022-12-16

**Authors:** Jodie Murphy-Oikonen, Karen McQueen, Ainsley Miller, Lori Chambers

**Affiliations:** 1 Lakehead University, Thunder Bay, ON, Canada

**Keywords:** sexual assault, police, police training, sexual assault investigation, police distress

## Abstract

Police officers play a central role in attaining justice for sexual assault survivors. Disclosing sexual assault is critical to attain justice and foster support, yet survivors often experience negative interactions when disclosing sexual victimization to the police. Police officers’ experiences investigating sexual assault have not been explored. Qualitative methods were used to explore the experiences of police officers who respond to reports of sexual assault. Semi-structured interviews with 20 police officers were analyzed in NVIVO software and uncovered four themes, (1) Lack of Sexual Assault Training; (2) Compassion for the Victim; (3) Investigative Process, and (4) Police Distress. The first-hand accounts of police officers uncover opportunities to improve police response to sexual assault and enhance the disclosure experience of survivors.

Police officers play a central role in attaining justice for sexual assault survivors as they have been deemed gatekeepers to the criminal justice system (Wentz & Keimig, 2019). However, police-reported sexual assaults are merely the “tip of the iceberg,” as they underrepresent a substantial global problem that is grossly underreported to law enforcement (Conroy & Cotter, 2017). Survivors disclose common barriers in reporting sexual assault to the police, including feeling it is too minor to report, shame, risk of retribution, and fear of being blamed or minimized (Spencer et al., 2017). Yet to attain justice, survivors must rely on police to investigate the sexual assault, charge the accused, and present the case to the Crown Attorney (Spohn & Tellis, 2019). In the absence of a charge made by the police, reported cases of sexual assault do not proceed through the justice system, thereby preventing closure for survivors (Morabito et al., 2019). Rotenberg (2017) found that one in five police reported sexual assaults proceed to court, and only 12% resulted in conviction. Given low conviction rates in sexual assault cases, police officers have received considerable public and media scrutiny, with national headlines reflecting biased attitudes among police officers and the failure of police to attain justice for victims (Doolittle et al., 2017).

Disclosing sexual assault is critical to attain justice and to foster support and healing for survivors (DePrince et al., 2020). However, survivors often report negative interactions when disclosing sexual victimization to the police including victim blaming, dismissive behavior, accusations of false reporting, and lack of empathy (Lorenz et al., 2019; Murphy-Oikonen et al., 2022) which leads to secondary victimization (Lorenz et al., 2019). Secondary victimization refers to the experience of trauma resulting from emotionally damaging, insensitive, blaming, and judgmental treatment of victims from the criminal justice system following the report of a crime (Haskell & Randall, 2019) and is often precipitated by adherence to rape myths.

Evidence suggests that many police officers ascribe to rape myths (Garza & Franklin, 2021; Shaw et al., 2017) which are stereotypical beliefs about who can be a victim or a perpetrator, and the conditions which constitute a sexual assault (O’Neal, 2019). These myths are false beliefs that often minimize victim credibility, blame the victim, discredit the circumstances, or shift culpability from the accused to the victim (Garza & Franklin, 2021; Shaw et al., 2017), thereby justifying the assault or denying that it occurred at all (Shaw et al., 2017). Likewise, a study by White and McMillan (2021) that explored the perceptions of police officers toward victims of sexual assault, found that many Canadian police officers disclosed suspicion toward victims of sexual assault. This is concerning as treating victims as suspects will inevitably lead to negative interactions, as survivors indicate that the quality of the disclosure experience to law enforcement is contingent on feeling respected and believed throughout the process (Henninger et al., 2020). The varying response from officers may be influenced by the feeling of responsibility to balance the dual role of protection of victims, while simultaneously protecting individuals who may be falsely accused of sexual assault (Ricciardelli et al., 2021).

While some police officers undoubtedly ascribe to rape myths, others may be perceived as blaming or dismissive of victims when they are in fact attempting to shield them from a criminal justice system that is re-victimizing based on victim-blaming beliefs, archaic practices, the burden of proof resting with the victim, defense counsel who interrogate victims, and excessively long timeframes in court (Spencer et al., 2018). According to Spencer et al. (2018), many police officers recognize the lack of support for victims through the criminal justice process, and the potential for a psychologically damaging trial. These officers espouse a need for procedural justice that supports the victim and empowers them to have a voice throughout the process as opposed to the successful outcome being contingent on a conviction.

In recent years, police organizations have taken steps to improve police response to sexual assault including on-site learning opportunities, Sexual Assault Review Committees (Ontario Association of Chiefs of Police, 2019), and changes in the Uniform Crime Reporting Survey to improve options for case clearances and data quality (The Canadian Centre for Justice Statistics, 2018), though the effectiveness of these initiatives is unclear (Moylan et al., 2017). Furthermore, Venema et al. (2020) found that while many police investigators have received training in sexual assault, training was variable in both quantity and quality and participants reported the victim-focused learning in areas such as interviewing to be merely “minimally helpful.” While these findings provide some evidence regarding police challenges in investigating sexual assaults, there is limited research that explores the voices of police officers and their experiences in sexual assault investigations. Thus, the purpose of this research was to explore the lived experiences of police officers in Ontario, Canada who respond to reports of sexual assault.

## Method

Descriptive phenomenology was used to explore the lived experiences of police officers in Ontario, Canada who respond to reports of sexual assault. Phenomenological methods are useful in revealing the structure of experience through a rigorous process. This results in a rich description supported by first-hand accounts of those who live it (Morrow et al., 2015). The context of Canadian policing is important, as justice in Canada is legislated through the provinces and police services operate at the federal (Royal Canadian Mounted Police), provincial, municipal (Robertson, 2012), and First Nations Police (FNPP) levels. FNPP is governed through a tripartite agreement with the federal and provincial government to provide culturally appropriate police services to Indigenous communities (Kiedrowski et al., 2017). Police involvement in Ontario is divided along geographical boundaries unless there is a special request from the Crown Attorney for a police service to become involved in a case outside of their jurisdiction. Police officers at all levels have considerable autonomy and discretion in decision-making (Robertson, 2012). Given the Canadian context of policing, recruitment of police officers may include participants from various levels of policing. The Research Ethics Board at an academic institution in Ontario, Canada approved all study materials and the consent process.

### Participants

Purposive sampling was used to recruit police officers from across the province of Ontario, Canada, between March 1st and April 4th, 2021. Recruitment strategies included: (a) social media advertising through Facebook and Twitter, (b) email recruitment notices to police associations in Ontario, Canada, and (c) word of mouth from police officers who participated in the study and shared the recruitment notice within their police detachment. The recruitment notices included the following two questions: (1) Have you investigated a sexual assault report? And (2) Would you like to share your perspectives on the process? The notice invited potential participants to contact the research team via cellular telephone or email to learn more about the research and express interest in participation.

The eligibility to participate in the study included: (1) English speaking current or former (retired) police officer in Ontario, Canada, (2) having previously responded to reports of sexual assault, and (3) the ability to participate in telephone interviews. A research assistant screened potential participants for inclusion and confirmed eligibility. Participants were subsequently provided with an information letter describing the purpose of the study, the risks of participating, confidentiality, the voluntary nature of the study, and the right of the participant to withdraw at any time. All individuals who inquired about the research met the inclusion criteria and were subsequently scheduled for interviews with the principal investigator (PI).

Nineteen current and one recently retired Ontario police officer contacted the research team to inquire about the research. All 20 participants met the inclusion criteria, provided verbal consent, and participated in an audio-recorded interview with the PI. Most participants were male, Caucasian, married/common law, heterosexual, and had postsecondary education. The smaller number of female participants (*n* = 5, 25%) is consistent with gender representation in policing. Furthermore, the sample included participants with a diverse range in age, officer rank, and years as a police officer. See Table 1 for participant characteristics.

**Table 1.** Participant Characteristics.

Gender (%)		Marital status (%)	
Male	15 (75)	Married/common law	16 (80)
Female	5 (25)	Single	4 (20)
Current age (%)		Highest level of education (%)	
<30	1 (5)	Highschool	2 (10)
30–49	11 (55)	College	4 (20)
>50	7 (35)	University	12 (60)
		Graduate	2 (10)
Ethnicity (%)		Officer rank (%)	
Caucasian	16 (80)	Constable Sargent	5 (25)
Indigenous	3 (15)	Staff Sargent	6 (30)
African American	1 (5)	Detective (sergeant, staff sergeant, detective inspector)	6 (30)
Sexual orientation		Member of sexual assault response unit	
Heterosexual	19 (95)	Yes	10 (50)
Gay	1 (5)	No	10 (50)
Police organization working for (%)		Years employed as a police officer (%)	
Municipal police	15 (75)	≤10	8 (40)
First Nation’s police	5 (25)	11–20	4 (20)
		21–30	8 (40)
		Mean/standard deviation	15.8 ± 9.8

### Data Collection

At the outset of the interview, the PI reviewed the information letter, answered any questions, and obtained verbal consent to participate and be audio-recorded throughout the interviews. Informed consent was audio-recorded and documented by the PI on a consent form. Data collection was conducted by the PI and included a single telephone interview with each participant (*n* = 20) which lasted an average of 65 min each. Telephone interviews were chosen to ensure participant anonymity, and participants were provided the option to use their first name or a pseudonym throughout the process. All participants elected to use their first name and were assigned a participant number to represent them in oral or written materials of the study. To maintain anonymity last names were not recorded.

Interviews were open-ended and followed a semi-structured interview guide. The guide was developed by the research team through a comprehensive review of the literature and in consultation with a sexual assault review team (SART) in the geographic area of the research. The SART included community service providers and health care workers who specialize in sexual assault, a district attorney, and several representatives of law enforcement. Consultation with the research team was conducted to ensure that the interview guide utilized appropriate language, and that all areas of query were appropriately addressed throughout the process. The final interview guide was verified by the SART and the research team and addressed four distinct areas including the: (1) process of responding to sexual assault cases, (2) challenges and barriers in responding to sexual assault, (3) decision-making in sexual assault cases, and (4) police training in sexual assault. Following the interview, participants completed a demographic questionnaire comprised of baseline characteristics (i.e., race, education, sexual orientation, annual income).

### Data Analysis

To aid in the analysis, all interviews were audio-recorded and transcribed verbatim. Transcripts were entered into NVIVO version 12 for analysis. Colaizzi’s (1978) thematic analytic method was used to organize and analyze the data. The initial step in the analysis included all research team members (authors of the manuscript) independently reviewing the individual transcripts prior to group analysis as a means of familiarizing themselves with the data. The research team subsequently reviewed the transcripts collectively and engaged in the extraction of significant phrases by coding each individual interview in NVIVO (55 initial nodes). Consistent with Colaizzi’s (1978) method, a constant comparative process was then undertaken by the team to formulate meaning from the extracted statements and establish themes that reflected the collective experience of participants. Discussion and debate occurred between all members of the research team to ensure the findings were accurately analyzed. Finally, the primary author wrote a comprehensive thematic description of participant accounts that was represented by various participant quotations to ensure credibility of the findings. These written thematic descriptions were further verified by the remaining members of the research team to ensure consistency with the findings. No changes to the thematic descriptions were deemed necessary. Data saturation was achieved when new data was consistent with previous participant accounts; this occurred at approximately interview 17, however, the research team completed all interviews that were previously scheduled.

## Findings

Data analysis revealed four themes and one sub-theme that reflected the first-hand experiences of Ontario police officers who have responded to reports of sexual assault. These themes included: (1) Lack of Sexual Assault Training; (2) Compassion for the Victim; (3) Investigative Process; and (4) Police Distress. The third theme of the “*Investigative Process*” included a sub-theme of “*Discretionary Decision Making.*” To ensure anonymity of participants, direct quotations from police officers are represented with a participant number.

## Lack of Sexual Assault Training

Police officers expressed a lack of sexual assault-specific training that left them with feelings of inadequacy and a lack of confidence in their ability to do a good and thorough interview when responding to sexual assault reports; “*I was just thrown into the fires*” (009). Participants in this research indicated that their basic training was only thirteen weeks long followed by mentorship from a coach officer. Many officers disclosed little experience in police work or related fields, and often their careers began in their early 20s. They discussed the breadth of police work and the compressed nature of police training, noting “*you are really expected to be a jack of all trades, but you don’t have the training to be any of those things*” (007). The complexity of learning a vast amount of new material in a short period of time led to a sense of insufficient knowledge in important parts of their work, including responses to sexual violence. An officer with over 25 years of service spoke about basic training:

There is so much to learn right? As a new recruit, you are really learning everything; the laws, the use of force. . .they touch on a bit of everything. And obviously there is a focus in self-defense training, because that is critical for our survival, so we spend a lot of time with shooting and self-defense scenarios and the law part is a really small portion (019).

Following traditional classroom training, police officers receive mentorship from a coach officer for approximately 3 months. Participants had mixed views on the effectiveness of the mentorship training pertaining to sexual assault. Some officers valued the ability to learn from veteran officers and peers through direct observation and guidance when receiving a sexual assault report, “*I was fortunate enough to have one of my training officers had just finished a term as a detective constable in sexual assault, so that was very helpful in terms of taking proper statements from people*” (014), whereas others acknowledged that the negative attitudes and experience of the coach officer was of pivotal importance to their development as police officers:

There are so many officers, in communities that aren’t happy with things and unfortunately it can come out during coaching. It actually has a huge impact on the recruit, because they see, right out the gate, how to act. They don’t get to see what’s great about the police service, they’re just right away exposed to everything that is jaded and that unfortunately has an impact on who they become as officers down the road (002).

Officers indicated that the geographic location that they were assigned also has a dramatic impact on the development of skills in interviewing and sexual assault. For example, in communities with low call volumes, learning about sexual assault was lessened, “*unfortunately by pure luck of the draw you could go through your entire training period and never even respond to a sexual assault investigation*” (016). One participant expressed the challenges of learning from the coach officer with limited experience:

We now have coach officers that are training new officers that have only been on the job for like 3 to 4 years themselves and have never done anything but front-line policing. So, their only perspective is wearing a uniform, driving a police car and doing initial investigations. They have never done in-depth criminal investigations. They don’t have a lot of experience in court, so as a result our young people don’t get trained as well (016).

Officers stated that they often learned on the job through observation, trial and error, making mistakes, being reprimanded by lawyers, or simply by “*flying by the seat of your pants*” and relying on your own judgment.

The majority of participants stated that sexual assault and victim interview skills training were “*very, very brief*,” “*very minimal*,” and “*inadequate.*” They disclosed that the content related to sexual assault was “*lumped together with other things*” and did not include important areas such as victim interviewing, the impact of trauma, how to set up an interview room, how to ask uncomfortable questions, how to deal with difficult emotions, or how to interview children. A veteran police officer recalled how he had to rely on his own skills due to the lack of training in how to interview victims of sexual assault, “*I didn’t have the skills, I didn’t have any. So, I was just using my own skills. You go to* [police college] *and take one course, you are done forever*” (013). Many of the participants spoke about the assumption during basic training that sexual assault investigations would be transferred to an investigator with increased training and skills in investigating sexual assaults, “*sexual violence would normally be reassigned to a more senior officer or detective to follow up on*” (018). However, not all police services have specialized investigation units, particularly among rural officers or detachments with human resource limitations. Additionally, even when investigators are available in a larger detachment, the initial reports are often taken by a patrol officer with limited knowledge and experience in interviewing or sexual victimization. Yet, participants acknowledged the importance of the initial interview to the investigation. An officer with over 20 years of experience spoke about his frustrations when sexual assault investigations could have been improved thereby resulting in less favorable outcomes for victims,

If you have something important in your workplace, you are going to have a tenured, experienced person dedicated to doing that. We don’t always have that control, so sometimes these negative experiences [between victim and police] are just a fact of who is available, and they just happen to be young and inexperienced. It is not necessarily about dropping the ball, or not knowing, they just don’t have the experience (015).

Despite police officers wanting to improve their skills in sexual assault interviewing and investigations, participants consistently expressed difficulties in accessing additional training. Many expressed a lack of access to sexual assault training given the limited number training spots available. Several participants indicated that larger detachments attain seats faster than smaller detachments, and priority is given to police officers entering an investigator role:

I’ve put my name into the Scene of Crime Officers course every year for the last 6 years. . .But, when the time came, we had a new detective that started in the crime unit. And because we were only allotted one seat, I automatically got bumped from it because they’re obviously in the line of work that requires it immediately, right? (003).

Furthermore, some participants were stationed in small remote communities that only had one officer in the community at a given time. They struggled with knowing that they required further training in sexual assault but leaving the community for training would cause considerable challenges in providing effective service. A rural police officer disclosed the difficulties in attaining training, “*the nightmare is in trying to get trained for uniform officers now. That means you are pulling them off the road for the 12-hour shift, then that platoon is short, I mean, it is a real balancing act*” (012). Geographical challenges also included increased costs associated with substantial distance to the nearest training opportunity.

Participants who were able to complete extra training in sexual assault and investigations were typically trained many years into their careers, for example, 8 to 10 years following basic training, “*to get a course as a front-line officer especially in the first couple of years, it just doesn’t happen*” (016). While those who received extra training found it beneficial, they acknowledged that some of the sexual assault calls they received early in their careers could have been vastly improved had they been provided with the training sooner.

## Compassion for the Victim

The police officers in this study expressed genuine care and compassion for survivors of sexual assault. They acknowledged the difficulties that survivors face in deciding to tell their stories to strangers in hopes of attaining justice and felt moved that people trusted them when they were most vulnerable, “*it is an honour in many regards that people have that much faith in you*” (003). Participants did not take their role lightly and worked diligently to gather as much evidence as they could to corroborate the survivor’s case to present the best case to the crown. Police officers expressed acute awareness of the difficulty of their questions during the investigation, and often worried that these questions would be perceived as blaming or dismissive. However, sensitive questions were necessary to develop the timeline of a case, or to collect any supporting evidence. Participants expressed concern regarding revictimizing the survivor and were very cautious in how they approached the victim interview. Several participants spoke of the need to be transparent during the investigation to ensure that survivors understood the motivation behind their questions. “*I found the challenges is them not understanding why we are asking certain questions, but the answer to that for me is just to provide them with that information as to why we are doing what we are doing*” (011). The police consistently expressed a desire to be victim-centered and cared genuinely about ensuring that the survivor was supported to the best of their ability. For example, one of the police officers expressed concern for the victim, “*as police, you know something happened to them so we need to take care of them when we could. If we are not laying a charge and the police just walk away, what happens to them after that?*” (013). The police officers expressed victim-centric values which included empathy, transparency, autonomy, and honoring women’s voices.

Participants noted the importance of compassionate and sensitive communication with survivors of sexual assault. Many participants spoke of “*being human*” or “*showing humanity*” and “*being real*” when speaking with a survivor of sexual assault. One of the officers spoke about how the communication during the initial contact with a survivor is essential to create an atmosphere of trust and set the survivor at ease:

It is a difficult experience for them to have to re-live and talk about anyway. Then, compound that by the fact that I am a complete stranger to them. So, I find the first difficulty is building that rapport, letting them know basically I am doing a job. And then the first challenge I find is truly showing them that I care, that I am not just a police officer who is taking your statement and then doing the paperwork (011).

The police officers in this study empathized with survivors and demonstrated a commitment to helping them through the case. “*If a police officer can just show, some calm empathy, some confidence in what they are doing, supportiveness, you can at least get the initial part of the investigation started*” (001). Some discussed the need to create a comfortable environment and to remove use of force equipment (i.e., Gun, vest) as a non-verbal communication strategy to align with the survivor while they disclose difficult information. Others spoke of the need to develop rapport, break the ice, actively listen whereby the survivor speaks freely and uninterrupted, and generally put the survivor at ease. A veteran officer expressed how he felt that knowledge of the process gives the survivor a sense of control or autonomy to make decisions:

I honestly think it is in your delivery, and that’s where I think a lot of police officers have failed. I think it is important that they understand that I believe you and I am here to move forward with this. But, as a disclaimer, just understand these are the challenges associated with it. I think it is how you communicate and not only in your approach and your tone and your choice of words, but just in your general demeanor (015).

Communication was not limited to the initial report. Participants also disclosed making efforts to keep the survivor informed throughout the process as much as possible. “*I know for me personally and for my detachments I look after, we at least try to give them* [survivor] *an update regularly, so they don’t feel like they are forgotten about and nothing is happening*” (009). While it was acknowledged that time and resources were barriers to continuous communication, police officers expressed the importance of ongoing follow-up with survivors, so they know where they stand. They also expressed the importance of communicating to survivors that their doors are always open and if something new emerges, they can come and talk to them at any time.

All police officers expressed feelings of wanting to protect survivors of sexual assault from a system that is often emotionally taxing and doesn’t always result in conviction. Participants indicated that the reference point for many survivors is often grounded in media cases whereby the assault happens, the report is made, the accused is charged and convicted expediently. In reality, cases may not be heard in a court room for a couple of years, which may interfere with the survivor’s healing process. While they want to support victims in seeing their case through the court process, they feel protective of survivors and their mental health and well-being. To protect the survivor from being blind-sided or interrogated on the stand many police officers described how they would discuss potential difficulties the survivor may experience moving forward. However, they expressed concern that conversations which are intended to be protective may be perceived as “*talking them out of*” the process. One officer articulated the delicate nature of these conversations:

I am not trying to talk somebody out of moving ahead, because I would quite bluntly say if you would like to proceed, by all means, we can do that. But I always felt it necessary to explain the associated challenges and how hard of a process it is going to be. My motivation is always just to be transparent because I kind of felt like we were feeding them to the wolves’ sort of thing. Because you come in, take the statement, lay the charge and everybody thinks everything is great, then they have all these hurdles along the way. So, many times people say I had no idea I would have to go through all this. (015)

Police officers also protected the victim by taking them to the hospital for rape kits and involving victim services following the assault. “*You could contact victim services and they would offer counselling, whatever they needed at that time. It was an option for them, just so that at the end of the night we weren’t just dropping them off at home alone*” (006). Participants expressed that they felt compelled to ensure that victims were cared for and well-informed so that they could reclaim control and have the autonomy to make decisions about their cases.

## Investigative Process

Police officers in this study spoke openly about some of the challenges early in their careers due to a lack of knowledge of trauma, re-victimization, and sexual assault investigations overall. Although all participants were officers who respond to sexual assault in Ontario, Canada, there was considerable variability in the investigative process depending on the jurisdiction in which the officer worked. In smaller communities and rural areas, many officers worked in isolation, with decentralized supervisory support available over the phone or on occasional community visits. In these communities, the officers were early in their careers and were responsible for responding to and investigating sexual assault cases without a specialized unit available to investigate. In larger cities, patrol officers often gathered “*tombstone information*” (participant described this as a term used to define information on a tombstone i.e., name, date of birth) and secured the scene and immediate evidence, while the investigation was the responsibility of the sexual assault division or major crime unit. For example, a police officer who worked in a remote community fairly independently described the differences in practices with larger centers, “*some of the bigger organizations, Ottawa police, Toronto, you know, they might have very specific sections to deal with specific issues; and we don’t have that*” (003). Conversely, an officer from a larger city stated, “*sexual assault will go straight to our crime unit, and then they will assign a detective to that, then the detective will reach out and arrange a time for an interview*” (013).

In addition to geographic differences in police response to sexual assault, police officers varied in their understanding of the process they should follow in sexual assault investigations. Those who felt confident in their role in sexual assault investigations disclosed that they understood standard operating procedures, who to involve in the investigation, the need to create a comfortable interview environment and video/audio tape interviews, involve healthcare professionals to complete rape kits, and follow up with survivors. Participants who were less confident in their role found the standard operating procedures to be vague. For example, while some participants “*always*” interviewed the accused, others “*never*” interviewed the accused. An early career officer described the vague operating procedures in sexual assault cases, “*we have standard operating procedures and um, . . . we have one on sexual assault and it’s just written in general terms. There’s nothing in there that says, when we get the report, interview victim then interview accused*” (004). These variations reflect challenges in investigations.

Additional inconsistencies in practice included the involvement of the supervisor in sexual assault cases. For some participants, the supervisor was notified automatically of any sexual assault investigations, some organizations had supervisors oversee all sexual assault cases, while other police officers contacted their supervisors as needed throughout the investigation. Overall participants found the support of supervisors to be critical to learning and support on the job. “*I think that the multiple eyes and the supervision is a key for sure*” (016). Though the use of consultation varied across participants, all valued the ability to “bounce” issues off of others in sexual assault cases. These consultations were not a standard; however, participants spoke of consulting with the crime unit, veteran officers, the crown, expert civilians, or their partner when responding to sexual assault reports.

Police officers disclosed that responding to and investigating sexual assaults is a very complicated process. Unlike other types of crimes where witnesses or video evidence is available, sexual assaults often occur in private with less opportunities for corroborating evidence. Participants indicated that there is a lot “*going through your head on what you can and can’t do*” (008) when investigating sexual assault, and while many questions are necessary to attain information in support of the case, “*you are basically trying to get initial information without getting too much information, without making them kind of have to re-live it*” (010). Participants acknowledged that the experience of both trauma and embarrassment often limit the information that is provided to the police by a survivor. At times, due to broken memories or the shame of disclosing private details, survivors’ stories change or are incomplete. This necessitates further questions from police to corroborate the account or build a case, although the questions are at times interpreted as doubt by the survivor. One officer disclosed the complicated nature of evidence collection,

We know there are three sides to every story. We know that there is stuff that the survivor or the complainant isn’t telling you because they may feel self-conscious about that information or they may just not remember because of trauma. And you know that the accused has a version. And right in the middle is exactly what happened, which is what you are trying to do right, take the two stories and put them together and have a plausible timeline and a plausible event chronology (008).

### Discretionary Decision-Making

All participants spoke of the complexity of decision-making in sexual assault cases and that determining reasonable grounds is very subjective. In the absence of evidence, the decision differs depending on the officer’s interpretation and discretion. One participant discussed how discretion differs by each individual officer:

It is my discretion whether I have chosen [the statement] to be compelling or not. And, what I may think is compelling from one person, the other investigator may not. So, there is nothing written in black and white saying this is what the definition of a compelling statement is; that becomes discretionary on the individual investigator (011).

Police officers indicated that decisions to charge the accused are not taken lightly. Participants rely on the victim’s statement and spoke about the “*believability*” of the report or whether the victim’s statement was “*compelling.*” An early career police officer stated that in the absence of evidence, he will often lay a charge based on the victim’s statement even if it may not result in a conviction, simply because he believes the victim. When asked how he makes the determination he stated: “*if she can give you the location, the time it happened, the date it happened, the circumstances, the story makes sense, that might be your reasonable grounds in a sexual assault, because of that fact that we don’t really require corroboration*” (010). However, officers indicated that they often seek support from their colleagues and supervisors when the decision to charge is particularly complex.

Though reports of sexual assault do not require corroboration to charge, police officers indicated that sexual assault cases often lack evidence, and result in a “he said–she said” scenario. The lack of evidence makes determining reasonable grounds to charge challenging for officers, however, they work diligently to follow the evidence to the best of their ability, as the evidence assists in broadening the victim’s statement. Yet the victim’s statement may be the only evidence to go on, “*95% of the time, the majority of the evidence we have to go on is the statement of the survivor*” (011).

While some officers indicated that the Crown Attorney does not influence their decision to charge, many participants expressed that the Crown Attorney and the court process do influence their decisions as they worry about the justice process and outcomes for survivors. They expressed that the system often fails survivors of sexual assault in that the Crown Attorney may not take the case to court due to lack of evidence, and if they do, the lengthy process and revictimization experienced from defense attorneys can be extremely emotionally damaging. An officer expressed her concerns for survivors who go to court, “*you are going to get into these long-drawn-out court cases that take forever to get to and you know the victim gets put up on the stand and just berated by the defense*” (020). Since the wrong decision could be damaging to either the survivor or the accused, the discretion to charge in a sexual assault case is given considerable thought. One officer spoke of the significance of their decision:

You just want to do the right thing. . .At the end of the day, I don’t think a lot of people realize that, like we are just making a really big, important decision with the information that we have. It’s kind of scary to think that you might make a wrong decision, but I don’t know what the alternative is? There is no fly on every wall in every room (010).

Participants also spoke about their observations whereby some officers make biased judgments before even speaking to a sexual assault survivor. These judgments are concerning given the autonomy of police officers in discretionary decision-making. Police officers were often challenged in an environment where some officers blamed victims, and rushed investigations or embraced opportunities to close a sexual assault case prematurely. “*I think a lot of people just; a lot of people don’t put a lot of work into it. So, they take a statement, and they say ok, I don’t really believe her*” (019). Although the culture is constantly changing and improving, participants described the impact that police attitudes and discretion in investigation can have on the case outcome. They stated that questioning these attitudes and challenging mishandled cases was “career suicide,” especially for early career and female police officers.

Police officers stated that many officers within their detachments ascribe to rape myths and are dismissive of victims which impedes sexual assault investigations. Disbelief of victims from some officers may stem from “*cynical attitudes*” based on people lying frequently to the police. These cynical attitudes permeate throughout the organization and become adopted by others, “*there was absolutely biases. I remember my coach officer saying you do not want one of these investigations, um, they are lying. Look for this when they are lying, it was not good*” (020). The culture of disbelief made sexual assault investigations “*the luck of the draw*” as some officers were very victim-centered, while others were victim-blaming. The officer who responded to a call was dependent on availability and the volume of calls at the time. An officer spoke of the officer’s disbelief and the impact on victims:

I remember one of them [police officer], they were congratulating each other in getting someone to confess that they weren’t raped and that they got caught cheating on their partner. They tried to call in a rape to cover it up. Now I look back at that with a different set of eyes and I am like holy shit, who knows what really happened, because they were painted with a broad brush before they even walked in the door (007).

The contradiction in the investigative efforts of study participants versus what they observed from other officers in the environment exemplifies the subjective interpretation of sexual assault reports and the use of police discretion in laying charges and case closures.

## Distress

Participants expressed fear, anxiety, and embarrassment as they tried to fulfill their role in sexual assault cases effectively. One participant stated, “*I remember being so terrified of the fact that they told me I had to capture it on video and, number one, I didn’t know how to work the video camera*” (002). Another participant expressed his worries when responding to a sexual assault call with very little experience and trying to do the right thing for the victim,

When I knew I was going to this woman’s house and she had probably just had the worst night of her life; walking into her house and seeing her in her bedroom curled up in a ball crying, it is something that I will never forget. Again, I may have been on the job a year, maybe two and it was just, it was something that I had never experienced before and I didn’t feel that I had the confidence, the training at that time to really help her. I felt completely unprepared (006).

The victim-centered approach of the police officers led many of them to experience distress during their investigations of sexual assault cases. Participants stated that police are often perceived as cold and uncaring. However, the human element of policing impacts them throughout the investigation and for years to come. A participant who was a police officer for 22 years spoke of the impact of public perception, “*I think people forget that officers are human, they have lives too, and all the same struggles that everybody else is going through*” (018).

Police officers expressed several incidents of distress when working on sexual assault cases. On a personal level, they worried about whether they were prepared or skilled enough to do victim interviews. They were distressed at the prospect of asking difficult questions and revictimizing the victim. They worried about public safety and about making wrong decisions and they struggled with having to deem a case as unfounded or not having enough evidence to proceed. In all instances, officers expressed that they wanted to do the best job they could for the victim and minimize the victim’s distress. One of the officers expressed distress with the process of the investigation and worry regarding what could have been done differently,

Could I have done more? Could I have written more warrants? What did I miss? or, What was the crux of the case, that you know, did I make an error procedurally that I didn’t know about in how I wrote a warrant or, how I collected evidence? Because no investigation is perfect and it never is, and the defense never attacks what actually happened, they attack the process you used to investigate it (018).

Other participants spoke about the worries they had throughout the investigative process regarding the approach they took, the questions they asked, and the possibility of revictimizing the victim. These concerns persisted years later when they reflected on their cases, “*until this day, I think back on it, and I wonder, did I approach this wrong?*” (001). Another police officer worried about the approach that he took when questioning a survivor:

Frankly, now it’s in the forefront of my mind. Do not do anything that is going to screw this case up, because that falls directly on me. It’s the thing that will potentially affect the victim. . . it’s going to re-victimize them 110% if I screw it up (003).

Participants also worried about the approach of other officers within their detachment. Given that the participants in this research were highly victim-focused, when the attitudes they observed in other officers led to mishandled cases, they often experienced distress. They stated that not all police officers are well-suited to sexual assault investigations. A female police officer explained the strain of working alongside judgmental officers:

I just happen to be in the office, and she [another officer] made a comment. She hadn’t even spoken to the victim yet and she said, well I guess someone was late for curfew and had to make up an excuse. And it was sort of like something that is made fun of and belittled and minimized. And, um, there is a belief that most sex assaults called in are fake or embellished, or the victim wasn’t clear on consent, or she was drunk, that’s another one too. Like if the person was drunk, don’t even bother. If you consumed any kind of drugs or alcohol or anything, I wouldn’t even bother [reporting] (007).

Other participants in the research experienced distress in knowing the lengthy and difficult legal process and the lack of services available to support the survivor following the assault. “*There is barely enough help for them to heal. . . it is dismal; it is disgusting, and the help for children is even worse*” (014). An officer spoke of the long-term impact of these cases as they worry about survivors, “*there is one case that was almost 15 years ago now. I would wonder from time to time whatever happened to her, you know, is that family still together? Is he still doing that? Has it gotten worse?*” (007). Participants clearly articulated their compassion for victims and wanting to do what was in their best interest. When case outcomes were not favorable or were unknown, they experienced distress as they worried for survivors’ well-being.

## Discussion

The first-hand accounts of police officers who have responded to reports of sexual assault provide an important contribution to the limited literature reflecting experiences in policing, relative to sexual violence. The participants in this research expressed a great deal of compassion for victims of sexual assault and were highly committed to seeking justice for survivors. However, they acknowledged challenges and barriers they face as police officers responsible for sexual assault investigations including a lack of sexual assault training and the difficulty of determining reasonable grounds. Many participants expressed both personal and professional distress in responding to reports of sexual assault as they worried about survivors’ well-being and felt the gravity of their role in the process. Overall, participant’s compassion for the victim and distress throughout the process reflect their commitment to justice in sexual assault cases.

The findings from this study provide a unique contribution as much of the existing literature predominately reflects police officer adherence to rape myths (O’Neal, 2019; Shaw et al., 2017) and media and public depiction of police officers who respond to sexual assault as uncaring and dismissive of survivors’ needs (Doolittle et al., 2017). This is in stark contrast to participants in our study who reflect a group of officers who are passionate about improving the disclosure experience for survivors of sexual assault. These officers reject rape myths and collectively endorse victim-centered values to treat each victim as unique and deserving of compassion and respect. These values highlight select officers as potential change agents within the existing police culture, relative to sexual assault. This is important as survivors who feel validated and supported by the police have an increased likelihood of sustaining involvement in the criminal justice system (Campbell et al., 2015), thereby optimizing opportunities for justice. Furthermore, the compassion of the police officers in the present research aligns with the needs of survivors who indicate that empathy, caring, sensitive communication, and belief in their victimization are pivotal to healing from sexual violence and avoiding secondary victimization (Murphy-Oikonen et al., 2022).

Our findings are consistent with existing research that initial police training is brief (3–6 months in North America; Cordner, 2019; Sloan & Paoline, 2021) and training specific to sexual assault is limited (Lathan et al., 2019). Criticisms of police training include a primary focus on laws and dynamics of the crime as opposed to the victim’s response or investigative process (Lonsway et al., 2001). Likewise, the consistency and efficacy of sexual assault training is reported as variable in both quantity and quality (Venema et al., 2020). However, other research has demonstrated that sexual assault specific training is effective in reducing police acceptance of rape myths, increasing knowledge of laws and guidelines, and improving understanding of trauma-informed practices among police officers (Campbell et al., 2020; Lathan et al., 2019). In our study, the lack of trauma-informed sexual assault training left many participants feeling inadequate or unprepared when they received reports of sexual victimization. These findings suggest that police organizations would benefit from expanding the timeline and content of existing training to ensure that all areas of policing are provided with adequate opportunities for learning. As the victim interview is a critical piece of evidence in sexual assault cases, police officer skill development is of high importance and should be inclusive of the needs of survivors (Haskell & Randall, 2019).

The importance of providing trauma-informed sexual assault training to all officers, regardless of rank, cannot be understated as current models of sexual assault training in policing reserve learning opportunities for investigative or veteran officers. Participants in our study revealed that many police officers who respond to sexual assault are early in their careers or reside in smaller jurisdictions where investigative units are lacking or unavailable. Researchers have found an association between a higher officer rank and positive attitudes and perceptions of sexual assault survivors compared to lower-ranked officers (Lorenz & Maskaly, 2018). In addition, officers from smaller police organizations are more likely to adhere to negative attitudes of sexual assault survivors than those from larger organizations. Therefore, improving understanding of trauma, and evidence-based sexual assault interviewing skills among all police officers may also improve the disclosure experience of survivors, enhance the survivor’s statement, and improve opportunities for justice in sexual assault cases.

Although enhanced training is a positive step toward improving the skill and compassion of police officers who respond to sexual assault, investigative challenges will remain. In particular, participants described a lack of standardization in responding to sexual assault and considerable discretion in determining reasonable grounds in sexual assault cases. According to Tasca et al. (2013), police officers’ decisions to arrest a suspect in sexual assault cases is highly influenced by the collection of forensic evidence. Yet, physical evidence is often limited in sexual assault cases, and the existence of evidence does not verify the issue of consent (Ali et al., 2019). To enhance the opportunity for evidence collection, guidelines or frameworks that assist officers of all rank to support survivors in attaining a medical exam and enhance understanding of various avenues for evidence collection, may increase officers’ confidence in the strength of their case. In addition, guidelines also indicate that a thorough investigation is required and some opportunities for evidence collection are noted (Ontario Association of Chiefs of Police, 2019), however, officers indicate that further clarity is required, and no two cases are exactly alike.

Nevertheless, since sexual assault cases often lack witnesses and evidence may be minimal, the victim interview is considered the most important source of evidence (Haskell & Randall, 2019). Thus, the interaction between the survivor and the police officer is critical to ensure that the disclosure experience is conducive to the survivor providing as much information as possible to assist in the investigation. These interviews can be strengthened with decreased acceptance of rape myths and increased compassion toward victims to create a comfortable environment and improve the quality of the victim’s statement (Haskell & Randall, 2019; Rich, 2019). Furthermore, sexual assault stakeholders have urged police officers to improve victim interviews through empowerment, supporting and believing victims, and understanding the limitations of disclosure (Ali et al., 2019). Since victim interviewing is complex, evidence-based models such as the PEACE model or Trauma-Informed Interviewing offer common principles (i.e., rapport building, eliciting a free narrative, and using open-ended versus closed questions), to serve the dual purpose of gathering robust statements and evidence from victims, while simultaneously being sensitive to their emotional needs and creating a comfortable interview space (Akca et al., 2021; Rich, 2019; Snook et al., 2010). These approaches have demonstrated promise in improving victim interviewing, although sustainability of the gains may be improved with additional learning or “booster” sessions (Akca et al., 2021).

Although policy on responding to sexual assault has been developed in many jurisdictions (Ontario Association of Chiefs of Police, 2019), implementation of policy in sexual assault cases is unclear. For example, the Canadian Framework for responding to sexual assault states that officers must respond swiftly to reports of sexual assault and approach survivors in a trauma-informed manner (Ontario Association of Chiefs of Police, 2019). Yet our findings indicate that officers do not learn about trauma-informed care in basic police training and only learn about the approach late in their careers, and previous research indicates that many survivors wait for long periods before police respond to their reports (Murphy-Oikonen et al., 2022). While national frameworks are invariably a positive step for improving police response to sexual assault, leadership must commit to ensuring understanding and implementation of policy among all police officers to enhance their role, as our findings suggest that officers find the policies vague and subject to interpretation.

The finding of police distress was evident throughout all themes in this research. Police officers experienced distress when a lack of training inhibited their confidence and skill in responding to sexual assault, distress was experienced based on their compassion toward victims, and it was also experienced throughout sexual assault investigations, particularly in determining reasonable grounds in a case and putting forth a solid investigation to the crown. The experience of distress in policing is well-established given the high-stress environment, the role of police as first-responders, and the cumulative impact of responding to traumatic incidents (Losung et al., 2021; Sheard et al., 2019; Velazquez & Hernandez, 2019) but it is seldom discussed with respect to responding to sexual victimization. Yet the officers in the current study experienced distress in investigating sexual assault, particularly based on their victim-centered focus. Losung et al. (2021) highlight the importance of empathy among police officers who respond to sexual assault and argue that many officers working in sexual violence attain professional satisfaction in assisting victims. Given that half of our participants were members of a sexual assault response unit, their commitment to ensuring justice for victims of sexual assault is evident. However, despite the compassion satisfaction gained from this role (Losung et al., 2021), our findings suggest that it is not without personal strain in officers’ efforts to support survivors and do their job well. The stressful nature of police work has a negative impact on officers’ overall mental health and well-being (Sheard et al., 2019), yet police culture often stigmatizes displays of emotion and the need for mental health support (Velazquez & Hernandez, 2019). The experience of distress highlights the need for greater organizational support for police officer’s well-being as they respond to sexual violence and challenges some of the recent negative discourse on police officers as uncaring, disbelieving, and dismissive when investigating sexual assaults. Supporting the mental health needs of police officers who are distressed when working with victims of sexual assault is important for officer’s mental health and may lead to improved support for victims.

### Limitations

Despite the strength of the first-hand accounts of a diverse sample of police officers who respond to sexual assault, the research is not without limitations. Our research was voluntary, and participants self-selected their involvement. All participants were similar in their commitment to justice and victim-centered approach to survivors of sexual assault. However, their self-perceptions of their approach to survivors might not match the perceptions of their approach from sexual assault survivors. Given the abundance of evidence that sexual assault survivors have experienced negativity and bias from police officers, the current victim-centered sample is not representative of all police officers. Furthermore, police officers were recruited from Ontario, Canada and their experiences may not reflect the realities of police officers from other jurisdictions, thereby limiting the generalizability of the findings. Finally, although interviews were robust, it is unclear if telephone interviews impacted the disclosures of participants.

## Conclusion

Public perception of sexual assault and sexual assault survivors is often informed by victim advocates, victims themselves, or public opinion, with little input directly from police officers. The current study reflects the first-hand accounts of police officers and highlights the complexity of the investigative process, the limitations of training in sexual assault, and the attitudes and beliefs of forthcoming police officers toward victims, often resulting in police officer distress. To attain justice in sexual assault cases, discourse in sexual assault must reflect the voices and experiences of all involved in the process. The contributions of police officers in this study strengthen opportunities for intervention to improve police response to sexual assault and ultimately strengthen the disclosure experience of sexual assault survivors.
